# Wonderful Intelligence of Dog

**Published:** 1889-04

**Authors:** 


### WONDERFUL INTELLIGENCE OF A DOG. HE REASONS, ACTS, AND SAVES LIFE.

A big jet black Newfoundland dog whose chief delight is to bark savagely at wayfarers, is the especial pride of the family of Mr. Smith of Belmont Belmont is a cluster of frame buildings near East 180th street. and Mr. Smith's house is a quaint one in the center of the group. The dog then a pup, turned up in Mr. Smith's yard four years ago, took a fancy to little Oscar Smith and little George Smith, and was allowed to stay. He was christened "Nit," and became the constant companion and playmate of the boys. Nit was always willing to be "sicked" on other dogs for the amusement of George and Oscar, and was equally ready to attack anybody who gave them offence. But Nit earned his greatest distinction on Sunday last. It is due to his efforts that the two little Smiths and the little boys of two other households in Belmont are alive to tell what a brave intelligent fellow Nit is.

On Sunday the little Smith boys started off with 13-year old Frank Wilson, 14-year old Willie Guggals, and another little fellow named Kehoe to have a good time the rest of the day. They tramped to the old Lorillard snuff mill on the Bronx River, and were romping about, when Nit ran up and joined in the fun. An old boat, twelve feet long, was fastened by a rusty chain to a stake, and all of the little fellows except Guggals climbed into it and were amusing themselves by rocking it when the chain broke and the boat drifted out from the shore. Hardly more than fifty yards down the river the water splashes over a dam and falls twenty feet on a mass of jagged rocks. There were no oars in the boat, and nothing that would serve in their stead. In the middle of the river the boat swung lazily around until the prow pointed toward the dam, and then it began slowly to drift down the stream.

Nit had stood on the shore with ears and tail erect watching the boat drift away, and apparently considering it a good joke. But when the boat began to move toward the dam Nit became ill at ease and ran barking and whining up and down the bank. The boys were thoroughly alarmed by this time, too. They cried out for help, and Nit telling them by a sharp, short bark to wait for aim, sprang into the water and beat his way with lusty strokes toward the boat, now dangerously near the dam. Nit swam right in front of the boat and tried to stop it with his body, but the current swung the stern around. Finding that that this wouldn't do, Nit swam around the boat twice, thinking very earnestly all the time. Having solved the difficulty, as he thought, he sprang up on the gunwale and seized it with his teeth. This lifted him so far out of the water that he couldn't swim. Then he let go his hold and went around the boat once more for another idea. He got it, and then the question arose how to convey it to the frightened youngsters. Nit swam close to the boat, and, sticking his head over the gunwale, looked imploring into the little Oscar's face and whimpered. Oscar misunderstood and thought Nit was tired and wanted to come in for a rest. He seized the leather strap that was buckled around the dog's neck and tried to lift him in. But Nit instantly dropped back into the water, and, pointing his head toward the shore, began swimming for all he was worth. Gradually the downward course of the boat was stopped. It swung around in answer to Nit's powerful legs, and slowly drew near the shore. It grounded within a few feet of the dam, and the boys sprang out as happy a lot of youngsters as lived. They started homeward on a run, with Nit barking and frolicking around them.

When a *Sun* reporter visited the Smith house yesterday afternoon Nit sprang up on the fence and dared him to come inside. Oscar ran out and was telling all about his adventure when Nit sprang up and with one sweep of his tongue washed Oscar's face. Mr Smith said that when he heard of the narrow escape of his boys he was so frightened that he had scarcely strength enough left to spank them.—*New York Sun*, March 15th.

